# Expression of Two Foreign Genes by a Newcastle Disease Virus Vector From the Optimal Insertion Sites through a Combination of the ITU and IRES-Dependent Expression Approaches

**DOI:** 10.3389/fmicb.2020.00769

**Published:** 2020-04-28

**Authors:** Lei He, Zhenyu Zhang, Qingzhong Yu

**Affiliations:** ^1^The Key Lab of Animal Disease and Public Health, Henan University of Science and Technology, Luoyang, Henan, China; ^2^Southeast Poultry Research Laboratory, US National Poultry Research Center, Agricultural Research Service, United States Department of Agriculture, Athens, GA, United States

**Keywords:** NDV, foreign genes, GFP and RFP, optimal insertion sites, co-expression, multivalent vector

## Abstract

Many Newcastle disease virus (NDV) strains have been developed as vectors to express a foreign gene (FG) for vaccine and cancer therapy purposes. The non-coding region between the phosphoprotein (P) and matrix protein (M) genes and the non-coding region behind the NP gene open reading frame (ORF) in the NDV genome have been identified as the optimal insertion sites for efficient FG expression through the independent transcription unit (ITU) and the internal ribosomal entry site (IRES) dependent expression approaches, respectively. To date, however, the majority of these NDV vectors express only a single or two FGs from suboptimal insertion sites in the NDV genome, obtaining various levels of FG expression. To improve the FG expression, we generated NDV LaSota vaccine strain-based recombinant viruses expressing two FGs, GFP, and RFP, from the identified optimal insertion sites through a combination of the ITU and IRES-dependent approaches. Biological assessments of the recombinant viruses indicated that the recombinants expressing two FGs were slightly attenuated with approximately one order of magnitude lower in virus titers when compared to the viruses containing a single FG. The FG expression efficiencies from the two-FG viruses were also lower than those from the single-FG viruses. However, the expression of two FGs from the optimal insertion sites was significantly (*p* < 0.05) higher than those from the suboptimal insertion sites. The expressions of FGs as monocistronic ITU were approximately 4-fold more efficient than those expressed by the bicistronic IRES-dependent approach. These results suggest that the NDV LaSota vector could efficiently express two FGs from the identified optimal insertions sites. The ITU strategy could be used for “vectoring” FGs in circumstances where high expression of gene products (e.g., antigens) is warranted, whereas, the IRES-dependent tactic might be useful when lower amounts of IRES-directed FG products are needed.

## Introduction

*Avian orthoavulavirus 1*, commonly known as Newcastle disease virus (NDV, used hereafter), is a member of the genus *orthoavulavirus* within the subfamily *Avulavirinae* of the family *Paramyxoviridae*^1^ (Amarasinghe et al., 2019). Virulent strains of NDV cause Newcastle disease (ND), a highly contagious viral disease of domestic and wild birds, threatening the poultry industry worldwide^2^ (Miller and Koch, 2013). Vaccination, combined with strict biosecurity measures, has been the recommended strategy to control ND for 60 years (Dimitrov et al., 2017). Human infection with NDV is uncommon, but people exposed to infected birds may experience headaches, flu-like symptoms, and conjunctivitis for 1–2 days (Swayne and King, 2003). However, there is no data to suggest that the infection can be transmitted human-to-human.

NDV contains a single-stranded, non-segmented, negative-sense RNA of approximately 15.2 kb in length that consists of six genes flanked by a 3′ Leader and 5′ Trailer in the order 3′-nucleocapsid protein (NP)-phosphoprotein (P)-matrix protein (M)-fusion protein (F)-hemagglutinin-neuraminidase (HN)-large polymerase (L)-5′ (de Leeuw and Peeters, 1999; Peeters et al., 1999; Samal, 2011). Two accessory proteins (V and W) are produced through the editing of the phosphoprotein messenger RNA (mRNA) (Steward et al., 1993). Unlike positive-stranded RNA viruses, the naked genomic RNA of NDV is not infectious. It must be encapsidated with the NP protein and associated with the P and L proteins, forming a ribonucleocapsid, to act as a template for RNA transcription and replication (Peeters et al., 1999).

Since the first reverse genetics system for NDV was established in the late 1990s (Peeters et al., 1999; Romer-Oberdorfer et al., 1999), the genomes of several NDV strains have been developed as vectors to express foreign genes (FGs) for vaccine and cancer therapy purposes. (Schirrmacher and Fournier, 2009; Kim and Samal, 2018). Most of these NDV vectors express only a single FG from an additional independent transcription unit (ITU), which is inserted into a non-coding region between native viral transcription units within the NDV genome (Yu, 2015). The level of FG expression can vary depending on the size of the insert, and more importantly, the location of the insert in the NDV genome. The optimal insertion site in the NDV genome for efficient expression of an FG through the ITU approach has been identified as the non-coding region between the P and M genes (Zhao et al., 2015). Besides the ITU approach, a bicistronic approach was developed using a second ORF downstream from an internal ribosomal entry site (IRES) to facilitate cap-independent translation (Zhang et al., 2015). The level of FG expression through this approach correlates well with the transcriptional gradient across the ordered NDV genes, being 3′ NP > P > M > F > HN > L 5′. Therefore, the optimal insertion site for efficient expression of an FG through the bicistronic IRES-dependent approach would be the non-coding region behind the 3′ proximal NP gene of NDV.

Efforts to express two FGs from a single NDV vector have involved the use of either two additional ITUs or a bicistronic ITU containing an IRES (Puhler et al., 2008; Ramp et al., 2011; Khattar et al., 2015; Hu et al., 2017, 2018). In most of these cases, the FGs were expressed from suboptimal insertion sites with various levels of FG expression. To improve the FG expression, in the present study, we expressed two FGs, the green fluorescent protein (GFP) and red fluorescent protein (RFP) genes as reporters, from the identified optimal insertion sites in the NDV genome through a combination of the ITU and IRES-dependent approaches. The data obtained from this study suggest that the NDV LS strain could efficiently express two FGs simultaneously from the optimal insertion sites for the development of multivalent vaccines and cancer therapeutics.

## Materials and Methods

### Cells, Viruses, and Nucleic Acid Isolation

HEp-2 (CCL-81; ATCC) and DF-1 (CRL-12203; ATCC) cell lines were maintained at 37°C, and 5% CO_2_ in Dulbecco’s Modified Eagle Medium (DMEM, Thermo Fisher Scientific, Carlsbad, CA, United States) supplemented with 10% heat-inactivated fetal bovine serum (FBS, Thermo Fisher Scientific), and antibiotics (100 U/ml Penicillin, 100 μg/ml Streptomycin, 0.25 μg/mL Amphotericin B, Thermo Fisher Scientific, Suwanee, GA, United States). The DF-1 cells were cultured in DMEM containing 10% allantoic fluid (AF) from 10-day-old specific-pathogen-free (SPF) chicken embryos for all subsequent infection experiments unless otherwise indicated. The NDV LaSota strain-based recombinant viruses, rLS, rLS-GFP, rLS-NP-I-RFP, and rLS/IRES-RFP/GFP, were generated previously (Hu et al., 2011; Zhao et al., 2014; Zhang et al., 2015; Hu et al., 2018). The modified vaccinia Ankara/T7 recombinant virus (MVA/T7) used during virus rescue to provide the bacteriophage T7 RNA polymerase was a kind gift from B. Moss, National Institutes of Health (Wyatt et al., 1995).

Viral RNA was isolated from the AF of NDV-infected chicken embryos and infected DF-1 cells using a QIAamp Viral RNA Mini Kit according to the manufacturer’s instructions (Qiagen, Valencia, CA, United States).

### Construction of rLS-Based cDNA Clones Containing the RFP and GFP Genes

All experiments in this study were carried out following the guidelines and protocols approved by the Institutional Biosafety Committee (IBC), US National Poultry Research Center, United States Department of Agriculture, Agriculture Research Service (USNPRC, USDA-ARS, Athens, GA, United States).

**FIGURE 1:**
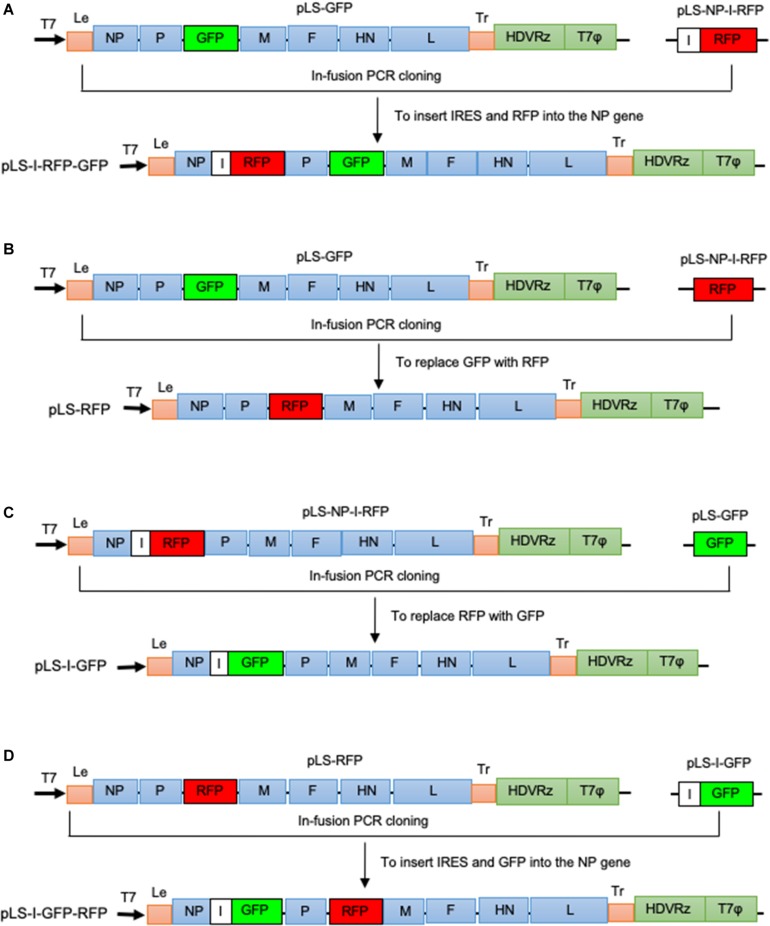
Schematic representation of NDV LaSota strain-based recombinant cDNA clones vectoring GFP and RFP (not to scale). The previously generated infectious clones, pLS-GFP (Zhao et al., 2014) and pLS-NP-I-RFP (Zhang et al., 2015; Zhao et al., 2014) were used as backbones to construct cDNA clones containing two FGs, GFP and RFP, in the identified optimal insertion sites using the In-Fusion^®^ PCR Cloning Kit following the manufacturer’s instruction (Clontech). **(A)** The IRES (I) sequence, together with the RFP gene amplified from pLS-NP-I-RFP, was inserted into the NP gene as a second ORF in pLS-GFP to create pLS-I-RFP-GFP. **(B)** The GFP gene in the pLS-GFP clone was replaced with the RFP gene amplified from pLS-NP-I-RFP, resulting in pLS-RFP. **(C)** The RFP gene in pLS-NP-I-RFP was replaced with the GFP gene amplified from the pLS-GFP clone, generating pLS-I-GFP. **(D)** The IRES sequence, along with the GFP gene, was amplified from pLS-I-GFP and inserted into pLS-RFP, resulting in pLS-I-GFP-RFP. A bold black arrow indicates the direction of the T7 promoter. Letters I, HDVRz, and T7Φ represent the IRES, Hepatitis delta virus ribozyme, and the T7 terminator sequences, respectively.

The previously generated infectious clones, pLS-GFP (containing the GFP insert between the P and M genes) (Zhao et al., 2014) and pLS-NP-I-RFP (containing the IRES and RFP insert as a 2nd ORF in the NP gene) (Zhang et al., 2015), were used as backbones to construct new cDNA clones, pLS-I-RFP-GFP, pLS-RFP, pLS-I-GFP, and pLS-I-GFP-RFP, as illustrated in Figure 1. Construction of these new clones was carried out by using an In-Fusion^®^ PCR Cloning Kit following the manufacturer’s instruction (Clontech, Mountain View, CA, United States). Primer sets used to amplify the GFP or RFP fragments and the linearized vector backbone were designed to contain a 15-nucleotide (nt) overlapping region of homology at their 5′ end (Table 1). All PCR amplifications were carried out by using *pfu*Ultra^TM^ II Fusion HS DNA polymerase (Agilent Technologies, La Jolla, CA, United States) and the paired gene-specific primers (Table 1) according to the manufacturer’s instruction. Briefly, the IRES sequence and RFP gene were amplified by PCR with a pair of specific primers (F1 F and F1 R, Table 1) from the pLS-NP-I-RFP clone, and inserted into the NP gene as a 2nd ORF in a linearized vector that was amplified from the pLS-GFP with one pair of specific primers (F1 Vec F and F1 Vec R, Table 1), creating pLS-I-RFP-GFP (Figure 1A). The GFP gene in the pLS-GFP clone was replaced with the RFP gene amplified from pLS-NP-I-RFP with two pairs of specific primers separately (F2 F and F2 R for RFP gene, F2 Vec F and F2 Vec R for the linearized vector, Table 1), resulting in pLS-RFP (Figure 1B). Similarly, the RFP gene in pLS-NP-I-RFP was replaced with the GFP gene amplified from the pLS-GFP clone with two pairs of specific primers (F3 F and F3 R for GFP gene, F1 Vec F and F3 Vec R for the linearized vector, Table 1), generating pLS-I-GFP (Figure 1C). The IRES sequence, along with the GFP gene in the pLS-I-GFP clone, was amplified and inserted into the NP gene as a 2nd ORF in the pLS-RFP clone with two pairs of specific primers (F1 F and F3 R for GFP gene, F1 Vec F and F1 Vec R for the linearized vector, Table 1), generating pLS-I-GFP-RFP (Figure 1D). All resulting cDNA clones were amplified with Stbl2 cells at 30°C for 24 h and purified by using a QIAprep Spin Miniprep kit (Qiagen).

**TABLE 1:** Primer sequences used in the study.

**Primer**	**Primer sequenced^c^**	**Primer name**
1^a^	gactgggggtattgaGCAAATTCCGCCCCTCTCC	F1 F
2^a^	gggttttgtccactaCTACAGGAACAGGTGGTG	F1 R
3^a^	atagttgtagccaccATGGCCTCCTCCGAGGACG	F2 F
4^a^	acggtagttacacacCTACAGGAACAGGTGGTGGCG	F2 R
5^a^	aaacacgatgataatATGGTGAGCAAGCAGATCCTGAAG	F3 F
6^a^	gctgggttttgtccaCTATCACACCCACTCGTGCAGGC	F3 R
7^b^	tagtggacaaaacccAGCCTGCTTCC	F1 Vec F
8^b^	tcaatacccccagtcGGTGTCG	F1 Vec F
9^b^	gtgtgtaactaccgtGTACTAAGCC	F2 Vec F
10^b^	ggtggctacaactatCAACTAAACTC	F2 Vec R
11^b^	attatcatcgtgtttTTCAAAGG	F3 Vec R

*^*a*^Primers 1-6 were used to PCR amplify the RFP, IRES-RFP, GFP, and IRES-GFP genes from the plasmid. ^*b*^Primers 7-11 were used to amplify or linearize the subclone vectors. ^*c*^Nucleotides shown in lower-case letters represent homology sequences with a vector backbone, which were used to facilitate the RE independent cloning using the In-Fusion^®^ PCR Cloning Kit (Clontech).*

### Virus Rescue and Propagation

Rescue of the recombinant viruses was performed by transfection of the recombinant cDNA clones and ancillary plasmids that express the NDV NP, P, and L proteins into MVA/T7 virus-infected HEp-2 cells as described previously (Estevez et al., 2007). Briefly, the HEp-2 cells were seeded on a six-well plate at 1 × 10^6^ cells/well and infected with MVA/T7 at a multiplicity of infection (MOI) of 1 to provide the T7 polymerase. A mixture of 2 μg of one of these four cDNA clones (pLS-RFP, pLS-I-GFP, pLS-I-RFP-GFP, and pLS-I-GFP-RFP), 1 μg of pTM-NP, 0.5 μg of pTM-P, and 0.1 μg of pTM-L was transfected into the MVA/T7 virus-infected HEp-2 cells using Lipofectamine^TM^ 3000 (Thermo Fisher Scientific) according to manufacturer’s instruction. At 72 h post-transfection, the rescued viruses were harvested by freeze-thawing the transfected cells for two times. The rescued viruses were amplified by inoculating 300 μl of the transfected cell lysate into the allantoic cavity of 10-day-old SPF chicken embryos and incubating the embryos at 37°C. After four days of incubation, the AF was harvested, and the presence of the rescued virus was detected by hemagglutination (HA) assay (Alexander, 1998). HA-positive AF was filtered through a 0.22 μm Nalgene Syringe Filter (Thermo Fisher Scientific) and amplified in chicken embryos for two more times. The AF was harvested from the infected embryos, aliquoted, and stored at −80°C as a stock. These rescued viruses were designated as rLS-RFP, rLS-I-GFP, rLS-I-RFP-GFP, and rLS-I-GFP-RFP, respectively.

### Pathogenicity Assessment, Virus Titration, Growth Kinetics, and Sequence Analysis

This pathogenicity study involving SPF chickens was carried out following the principles and recommendations of the Guide for the Care and Use of Agricultural Animals in Research and Teaching. The protocol was approved by the USDA-ARS, US National Poultry Research Center’s Institutional Animal Care, and Use Committee. Pathogenicity of the recombinant viruses was determined using the standard mean death time (MDT) and intracerebral pathogenicity index (ICPI) assays (Alexander, 1998). Titers of the recombinant viruses were measured by the standard HA test in a 96-well microplate, the 50% tissue culture infective dose (TCID_50_) assay on DF-1 cells, and the 50% egg infective dose (EID_50_) assay in 9-day-old SPF chicken embryos (Alexander, 1998). The growth kinetics of the recombinant viruses *in vitro* was determined using DF-1 cells. Monolayers of DF-1 cells were infected with the recombinant viruses at 0.01 MOI, respectively. Every 12 h post-infection, the infected DF-1 cells were harvested by freezing and thawing for two times and stored at −80°C until being tested. Viral titers were determined by the TCID_50_ assay on DF-1 cells for each time point in triplicate (Alexander, 1998). The mean titer of each time point of the viruses is expressed in Log_10_ TCID_50_/ml. The parental rLS, rLS-GFP, and rLS-NP-I-RFP viruses were included in the growth kinetics assay as controls. The nucleotide sequences of the rescued viruses were determined by sequencing the RT-PCR products amplified from the viral genome, as described previously, to confirm the sequence fidelity of the rescued viruses (Hu et al., 2011).

### Examination of GFP and RFP Expression

DF-1 cells were grown in 12-well plates and infected with the recombinant viruses at 0.01 MOI, respectively. Every 24 h post-infection, the appearance of GFP (green) and RFP (red) fluorescence in the infected cells was examined and digitally photographed using an inverted fluorescence microscope at 100× magnifications (AMG, EVOS fl, Grand Island, NY) with GFP and RFP specific filters, respectively.

### Quantification and Comparison of GFP and RFP Fluorescence Intensities

Monolayers of DF-1 cells in 96-well plates were infected with the recombinant viruses at 0.01 MOI, respectively, and incubated at 37°C in 5% CO_2_. The expressions of GFP and RFP were quantitated by measuring the green (GFP) and red (RFP) fluorescence intensities every 24 h post-infection using a Fluorescence Microplate Reader (BioTek, FLx800, Winooski, VT, United States) with a 485/20 excitation filter and a 528/20 emission filter for GFP, and a 540/35 excitation filter and a 600/40 emission filter for RFP. For comparison, the relative GFP and RFP fluorescence intensities measured from triplicate wells in two independent experiments were normalized to the highest intensity detected in the same experiment, which was set as 100%. The percentages of the GFP or RFP fluorescence intensities expressed by the recombinant viruses at different time points were plotted. The differences in the percentages of fluorescence intensities expressed by the two-FG viruses from the optimal and suboptimal insertion sites relative to those expressed by the single-FG viruses were compared and analyzed using the student *t*-test with a 5% level of significance (Microsoft Excel).

## Results

### Generation of the rLS Viruses Containing the RFP and GFP Genes

Based on the NDV infectious clones, pLS-NP-I-RFP and pLS-GFP, four full-length cDNA clones, pLS-I-RFP-GFP, pLS-I-GFP-RFP, pLS-I-GFP, and pLS-RFP, were constructed as illustrated in Figure 1. These clones contained the complete antisense genome of the NDV LaSota vaccine strain containing the GFP or RFP gene in the non-coding region between the P and M genes as an additional ITU and the IRES-RFP or IRES-GFP in the non-coding region in the NP gene as a 2nd ORF. The total length of cDNA clone in the pLS-I-RFP-GFP, pLS-I-GFP-RFP, pLS-I-GFP, and pLS-RFP plasmids is 17,370, 17,370, 16,494, and 16,062 bps, respectively, and all are divisible by six abiding by the “Rule of Six” (Kolakofsky et al., 2005). After co-transfection of the full-length cDNA clones and supporting plasmids into HEp-2 cells and subsequent propagation in SPF chicken embryonated eggs, the LaSota strain-based recombinant viruses containing either a single FG (GFP or RFP) or two FGs (GFP and RFP) in the optimal insertion sites were successfully generated. These rescued recombinant viruses were designated as rLS-I-RFP-GFP, rLS-I-GFP-RFP, rLS-I-GFP, and rLS-RFP, respectively. The nucleotide sequence fidelities of these recombinant viruses were confirmed by using sanger-based sequence analysis of the RT-PCR products of the viral genomes.

### Biological Characteristics of the rLS Viruses Containing the RFP and GFP Genes

**FIGURE 2:**
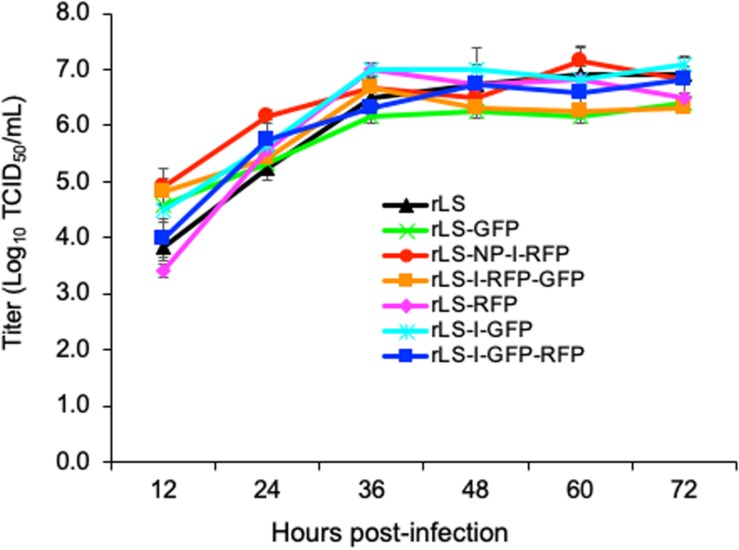
Growth kinetics of the recombinant viruses. DF-1 cells were infected with the indicated NDV viruses at 0.01 MOI. Every 12h post-infection, virus lysates were harvested. Virus titers were measured by TCID_50_ titration on DF-1 cells for each time point in triplicates from two independent experiments and expressed in mean log_10_ TCID_50_/mL with a deviation.

The rLS-I-RFP-GFP, rLS-I-GFP-RFP, rLS-I-GFP, and rLS-RFP viruses were examined *in vitro* and *in vivo* by performing the virus titration and the MDT and ICPI assays (Alexander, 1998) to evaluate the influence of the addition of the GFP and RFP genes on viral pathogenicity and growth ability. As shown in Table 2, the recombinant viruses containing either a single FG (GFP or RFP) or two FGs (GFP and RFP) were slightly attenuated with a longer MDT (>140 h) and a lower ICPI (0.0) compared to the parental rLS strain. In embryonated eggs, the titers of the recombinant viruses containing two FGs were approximately one order of magnitude lower relative to those of the parental LaSota strain and, more importantly, those containing one FG. Although the insertion of an additional FG did decrease viral yields, the recombinant viruses displayed comparative replication kinetics (Figure 2), suggesting that the insertion of either one FG (GFP or RFP) or two FGs (GFP and RFP) did not markedly influence the viral growth dynamics in DF-1 cells.

**TABLE 2:** Biological assessments of the NDV recombinant viruses.

**Viruses**	**MDT^a^ (h)**	**ICPI^b^**	**HA^c^**	**EID_50_^d^**	**TCID_50_^e^**
rLS	134	0.15	2048	3.16 × 10^9^	5.62 × 10^8^
rLS-GFP	140	0.0	2048	3.16 × 10^9^	1.78 × 10^7^
rLS-I-GFP	>150	0.0	512	6.81 × 10^9^	3.16 × 10^8^
rLS-I-RFP-GFP	>150	0.0	256	5.62 × 10^8^	1.78 × 10^7^
rLS-RFP	>150	0.0	512	4.22 × 10^9^	5.62 × 10^8^
rLS-NP-I-RFP	>150	0.0	2048	6.81 × 10^9^	3.16 × 10^8^
rLS-I-GFP-RFP	>150	0.0	256	5.62 × 10^8^	1.78 × 10^7^

*^*a*^Mean death time assay in embryonating eggs. ^*b*^Intracerebral pathogenicity index assay in day-old chickens. ^*c*^Hemagglutination assay. ^*d*^The 50% egg infective dose assay in embryonated eggs. ^*e*^The 50% tissue culture infective dose assay on DF-1 cells.*

### Co-expression of FGs by rLS-I-RFP-GFP and rLS-I-GFP-RFP

**FIGURE 3:**
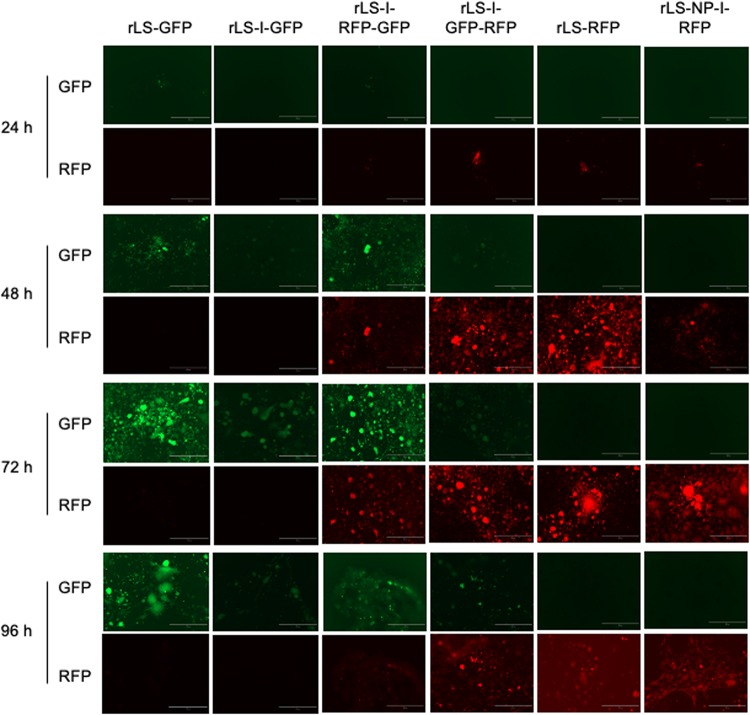
Detection of GFP and RFP expression from the recombinant virus-infected cells by fluorescence microscopy. DF-1 cells were infected with the indicated recombinant viruses at 0.01 MOI. Every 24h post-infection, infected cells were examined under an inverted fluorescence microscope at 100 × magnifications (AMG, EVOS fl, Grand Island, NY). GFP (green) and RFP (red) fluorescence from the same field of the infected cells was digitally photographed. The white bar represents 400 μm in size.

Expression of the FGs, GFP, and RFP, from the recombinant virus-infected DF-1 cells, was observed using an inverted fluorescence microscope. As shown in Figure 3, very few cells fluoresced at 24 h post-infection. As the infection progress, more and more fluorescent cells or fusion foci were observed, and the number of fluorescent cells or fusion foci and the brightness of the GFP and RFP fluorescence reached the peak around 72–96 h post-infection. As expected, rLS-GFP and rLS-I-GFP infected cells emitted only green fluorescence, whereas, the rLS-RFP and rLS-NP-I-RFP infected cells produced red fluorescence only. Both of green (GFP) and red (RFP) fluorescence were observed in the rLS-I-RFP-GFP and rLS-I-GFP-RFP infected cells. It appeared that the GFP and RFP fluorescence expressed from the additional ITU was much brighter than that expressed from the 2nd ORF in the NP gene. Whereas, the GFP or RFP fluorescence brightness expressed from the two-FG viruses (rLS-I-RFP-GFP and rLS-I-GFP-RFP) was slightly weaker than that expressed from the single-FG viruses (rLS-GFP, rLS-RFP, rLS-I-GFP, and rLS-NP-I-RFP). These observations indicated that the ITU approach was more efficient than the IRES-dependent method for FG expression, and the insertion of a 2nd FG adversely affected the 1st FG expression.

### Comparison of FG Expression Efficiencies

**FIGURE 4:**
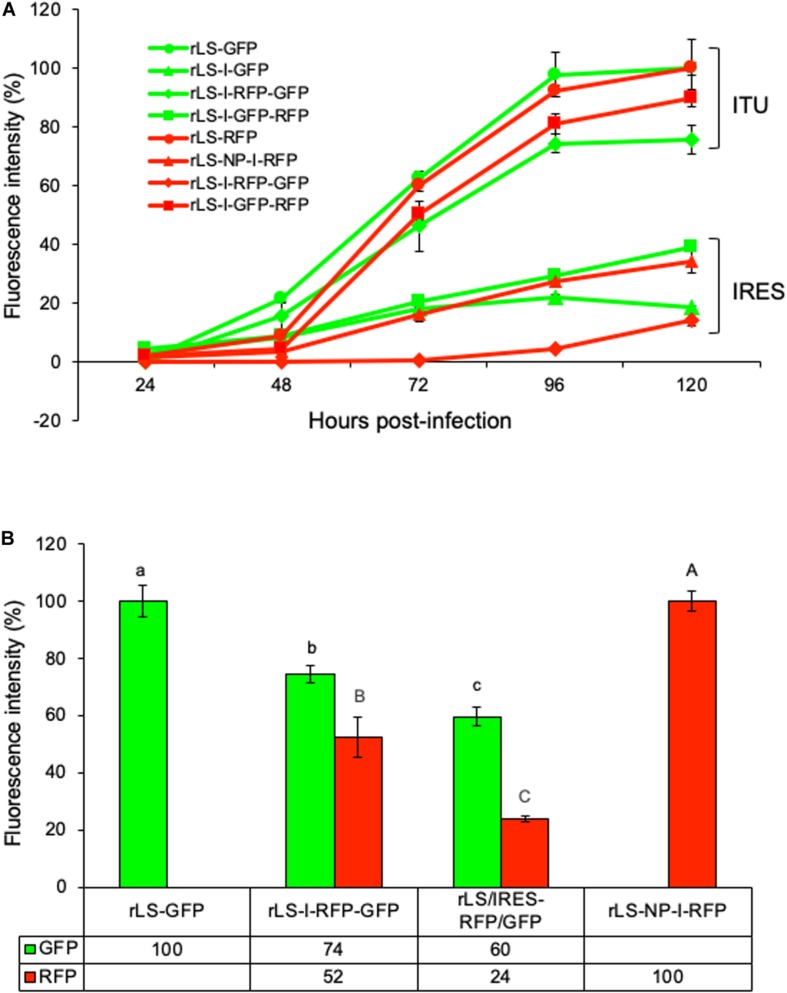
Comparisons of GFP and RFP expressed from different recombinant virus-infected DF-1 cells. DF-1 cells were grown in 96-well plates and infected with the indicated NDV recombinants at 0.01 MOI. Every 24h post-infection, GFP and RFP fluorescence intensities were measured by using Fluorescence Microplate Reader (BioTek, FLx800, Winooski, VT) in triplicate wells from two independent experiments. The highest GFP or RFP fluorescence intensity is deemed as 100%. **(A)** The percentages of the mean GFP and RFP fluorescence intensities respectively relative to their highest intensity at different time points post-infection were plotted. ITU and IRES denote the GFP and RFP expressed through the ITU and IRES-dependent approaches, respectively. Error bars indicate the standard deviation (SD) of GFP or RFP fluorescence intensity. **(B)** The percentages of the mean GFP or RFP fluorescence intensities respectively relative to their highest intensity were compared using the student t-Test with a 5% level of significance (Microsoft Excel). The lower-case letters (a, b, and c) indicate the significant difference (*p* < 0.05) of GFP fluorescence intensities and the capital letters (A, B, and C) denote the significant difference (*p* < 0.05) of RFP fluorescence intensities, between the indicated NDV recombinant virus-infected DF-1 cells, respectively. Error bars indicate the SD of GFP or RFP fluorescence intensity.

The fluorescence intensities of GFP and RFP were quantitated in the virus-infected DF-1 cells to compare the expression efficiencies of the FGs by the recombinant viruses through the different expression approaches. As shown in Figure 4A, the GFP and RFP fluorescence intensities were low during the first 48 h of infection; however, afterward, the fluorescence intensities increased rapidly and reached to the highest level (deemed as 100%) at 96–120 h post-infection. As a group, the GFP and RFP fluorescence intensities expressed by NDV recombinants through the ITU approach were approximately 4-fold more than those through the IRES-dependent method. The two-FG viruses (rLS-I-RFP-GFP and rLS-I-GFP-RFP) expressed 10-48% less GFP or RFP when compared to the single-FG viruses (rLS-GFP, rLS-I-GFP, rLS-RFP, and rLS-NP-I-RFP). However, as shown in Figure 4B, the rLS-I-RFP-GFP virus expressed significantly higher levels of GFP (*p* = 0.027) and RFP (*p* = 0.007) than the rLS/IRES-RFP/GFP, in which the I-RFP was inserted in the F gene as a 2nd ORF, and the GFP gene was inserted between the F and HN genes in the NDV genome (Hu et al., 2018). These results demonstrated that the ITU expression approach was more efficient than the IRES-dependent method for the FG expression. The expression efficiency of two-FGs by the NDV vector from the optimal insertion sites was significantly improved when compared to that from the suboptimal insertion sites, although the additional FG insertion adversely affected the overall FG expression.

## Discussion

Vaccinations have been proved to be the most cost-effective way of preventing and controlling animal infectious diseases, especially those caused by viruses. However, immunization strategies are facing many new challenges. Some of the vaccines used by the livestock farms had been developed several decades ago, while the vaccine targeted viral pathogens have evolved in farmed animals (Roohani et al., 2015; Nan et al., 2017; Yang et al., 2017). The mismatch of the antigenicity between the “old” vaccines and the currently circulating pathogens has frankly compromised the vaccine protective efficacy. To overcome the problem associated with this mismatch, researchers have developed viral vectors such as turkey herpesvirus, NDV, and fowlpox virus (Bublot et al., 2006; Esaki et al., 2013; Townsend et al., 2017; Kim and Samal, 2018) to deliver antigens that can match with the variants of currently circulating pathogens. Among these vectors, NDV has been demonstrated to be one of the most promising candidates for developing multivalent poultry vaccines (Dimitrov et al., 2017; Kim and Samal, 2018).

During the last decade, several NDV vaccine strain-based recombinants containing two antigenic components from a human or avian pathogen have been generated as vaccine candidates (Ramp et al., 2011; Khattar et al., 2015; Hu et al., 2017). Clinical trials with these vaccine candidates obtained various levels of protection against the targeted pathogens. Among these vaccine candidates, most of the antigens were expressed from suboptimal insertion sites in the NDV vectors through either two additional monocistronic ITUs or a bicistronic IRES-dependent expression approach. Although many factors could influence the protective immunity induced by the vectored vaccines, the expression efficiency of the FGs is undoubtedly the most important one. To improve the FG expression efficiency, in this study, we expressed two FGs from the identified optimal insertion sites in the NDV LaSota genome through the combination of the monocistronic ITU and the bicistronic IRES-dependent approaches. The data obtained here demonstrated that the levels of GFP and RFP expressed by the two-FG viruses from the optimal insertion sites were significantly higher than those expressed from the suboptimal insertion sites between the F and HN genes of the NDV genome. This finding proves that the LaSota vaccine is a promising vector that can efficiently express two FGs from the identified optimal insertion sites, which could potentially improve vaccine and cancer therapy efficacies.

In this study, we noticed that the titers of the NDV recombinant viruses containing two FGs were slightly lower (about 1 log lower) than those of the viruses containing a single FG. Obviously, the insertion of a 2nd FG into the NDV genome would increase the numbers of transcription units or the total length of the viral genome. It has been approved that the increase of the gene numbers and the length of the NDV genome would attenuate the virus replication, and reduce virus titers in the infected chicken embryonated eggs (Yu et al., 2013; Zhang et al., 2015; Zhao et al., 2015; Hu et al., 2018). The lower titers of the two-FG viruses may account for the decrease of the overall FG expression when compared to the recombinant viruses containing a single FG.

The data obtained in this study demonstrated that the monocistronic ITU expression approach was more efficient (about 4-fold) than the bicistronic IRES-dependent approach regardless of which of these two FGs to be expressed. This finding is in agreement with the previous report that the efficiency of the cap-dependent expression of 5′ proximal gene is significantly higher than that of IRES-dependent expression (Mizuguchi et al., 2000). Thus, it would be the first choice to express a higher amount of an FG product through the ITU approach. However, we would not recommend to express multiple FGs using multiple ITUs, because the insertion of ITUs in the NDV genome could attenuate their down-stream viral gene transcription and consequently decrease virus titers and the overall FG expression (Zhao et al., 2015; Hu et al., 2018).

In addition to the ITU and IRES approaches, a third method for the co-expression of FGs by an NDV vector was developed using the viral 2A self-cleavage peptides (Wen et al., 2015; Xu et al., 2018). Xu et al. (2018) generated a recombinant NDV co-expressing IL-15 and IL-7 through the incorporation of a 2A self-processing peptide into an IL-15/IL-7 fusion polypeptide and demonstrated that the modified NDV is a promising agent for cancer immunotherapy. The self-cleavage 2A strategy has a higher expression efficiency than the IRES method (Wen et al., 2015; Zhang et al., 2015), and can be used to express a similar or equal molar amounts of monomeric proteins for therapeutic purposes. However, there is a concern that after a self-cleavage of the expressed polypeptide, the upstream protein includes an extra short 2A peptide on its C-terminus (17–21 amino acids, depending upon the 2A sequence used), and the downstream protein contains a single proline residue on its N-terminus (Wen et al., 2015; Xu et al., 2018). There is a possibility that these extra amino acids may alter the antigenicity or biological functions of these foreign proteins, which might potentially affect the vaccine and cancer therapy efficacies.

In summary, the NDV LaSota vaccine strain-based recombinant viruses containing two FGs, GFP, and RFP, in the identified optimal insertion sites were slightly attenuated with about one order of magnitude lower in virus titers when compared to the viruses with a single FG and their parental rLS virus. The FG expression from the two-FG viruses was less efficient than those from the single-FG viruses. However, the expression of two FGs from the optimal insertion sites was significantly higher than those from the suboptimal insertion sites. The expression of FGs through the ITU approach was more efficient (about 4-fold) than that through the IRES approach. The results suggest that the NDV LaSota vaccine strain could efficiently express two FGs from the optimal insertion sites through the combination of the ITU and IRES expression approaches. The ITU tactic could be used for the expression of a higher amount of FG products, whereas, the IRES strategy might be useful to express a lower amount of FG products to meet the requirement for vaccine development or cancer therapy.

## Data Availability Statement

The datasets generated for this study are available on request to the corresponding author.

## Author ContRibutions

LH and QY conceived and designed the experiments, wrote the manuscript, and critically revised the content. LH and ZZ performed the laboratory experiments. All authors read and approved the final manuscript.

## Conflict of Interest

The authors declare that the research was conducted in the absence of any commercial or financial relationships that could be construed as a potential conflict of interest.

## Footnotes

1 https://talk.ictvonline.org/taxonomy/

2 https://www.oie.int/en/animal-health-in-the-world/animal-diseases/Newcastle-disease/
